# Supplementary figures and images for: Focal adhesion-derived liquid-liquid phase separations regulate mRNA translation
Source: eLife. 2025 Jun 26;13:RP96157. doi: 10.7554/eLife.96157 (PMC12201949; doi:10.7554/eLife.96157)

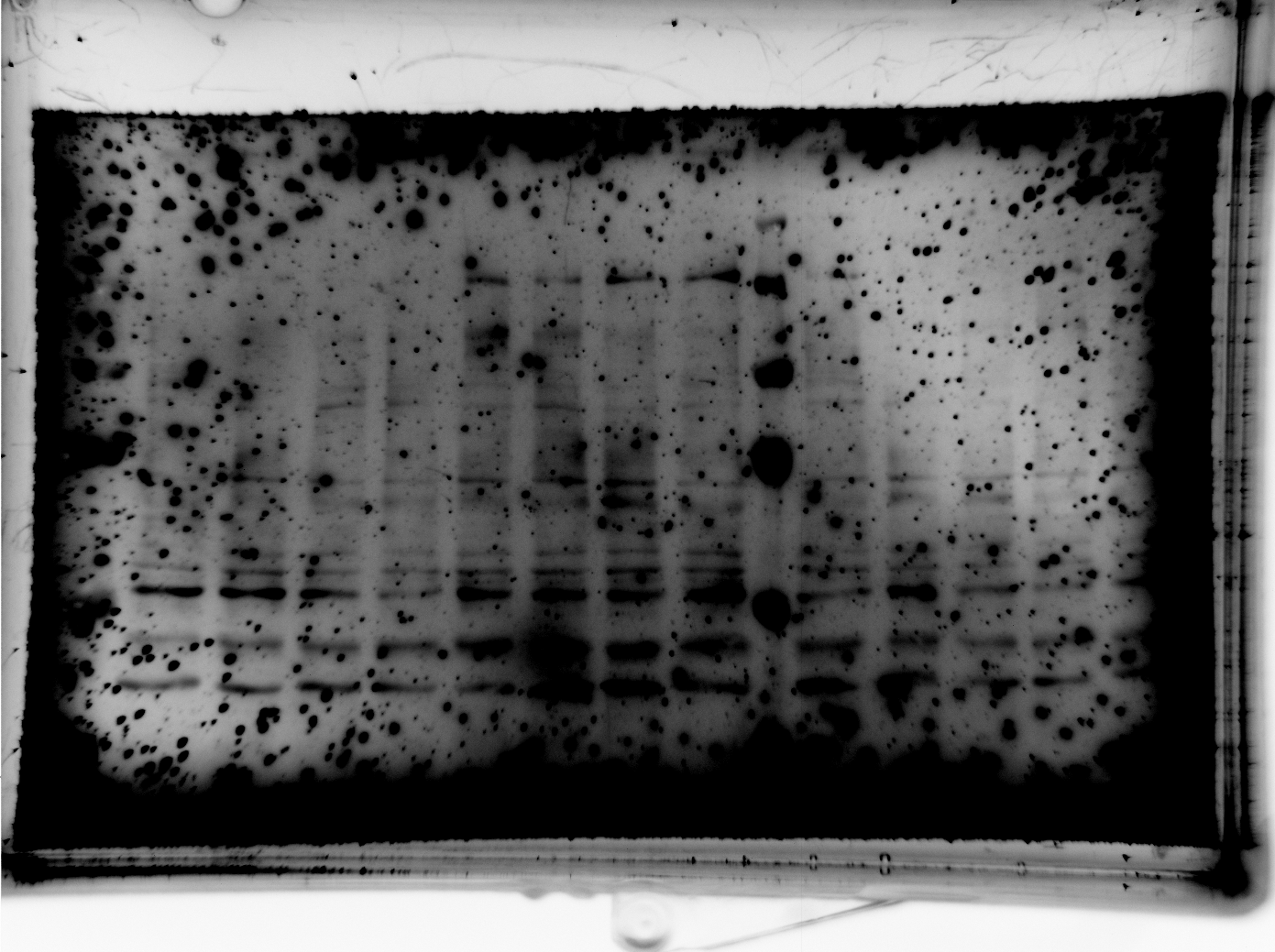

Supplement: Figure 1—figure supplement 1—source data 1. [file elife-96157-fig1-figsupp1-data1.zip › Figure 1- figure supplement 1- source data 1/Figure 1- figure supplement 1- source data 1.tif]

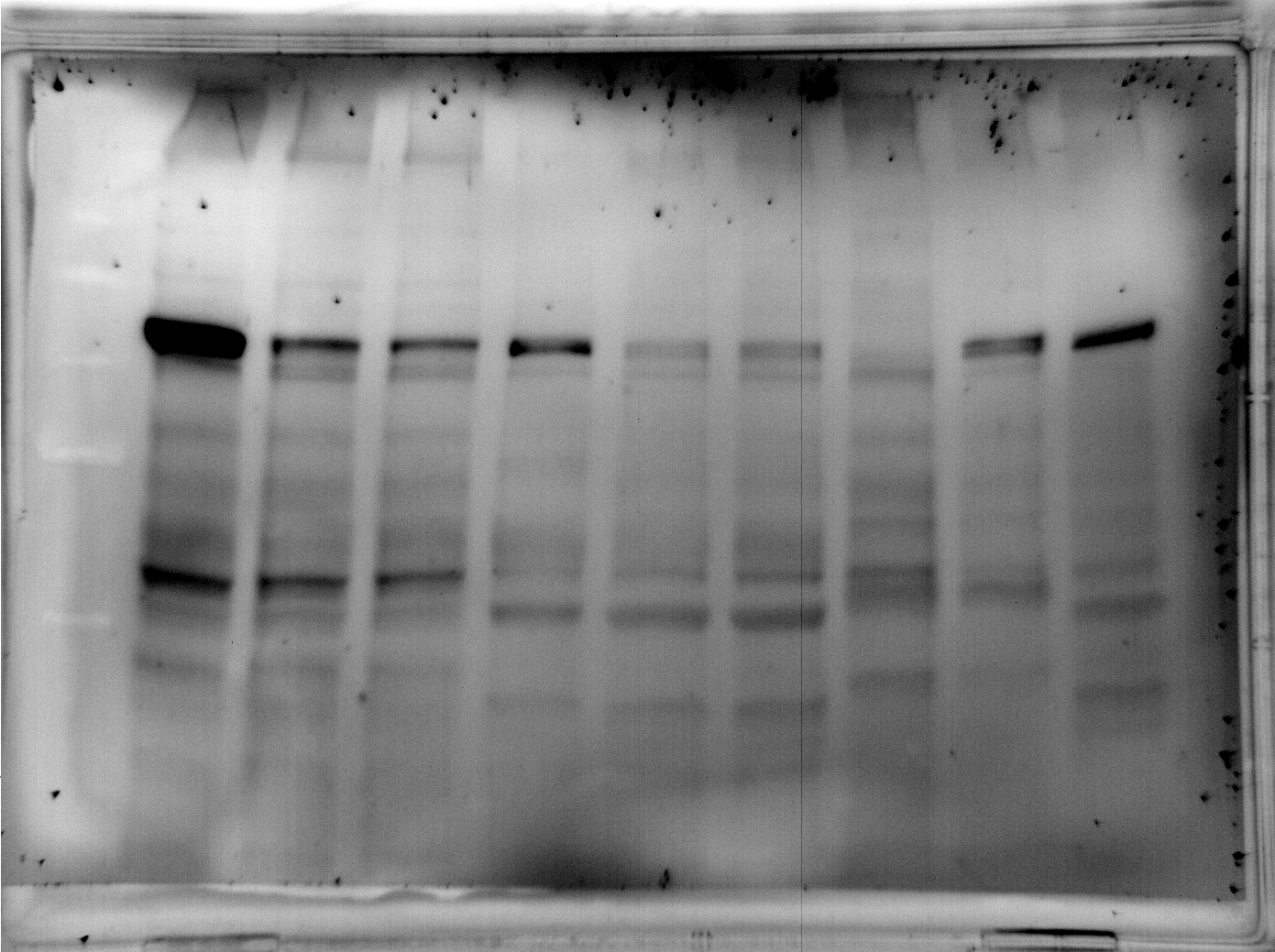

Supplement: Figure 1—figure supplement 1—source data 2. — The membrane was first developed using p130cas antibody (A) and then using GAPDH antibody (B). [file elife-96157-fig1-figsupp1-data2.zip › Figure 1- figure supplement 1- source data 2/Figure 1- figure supplement 1- source data 2 A.tif]

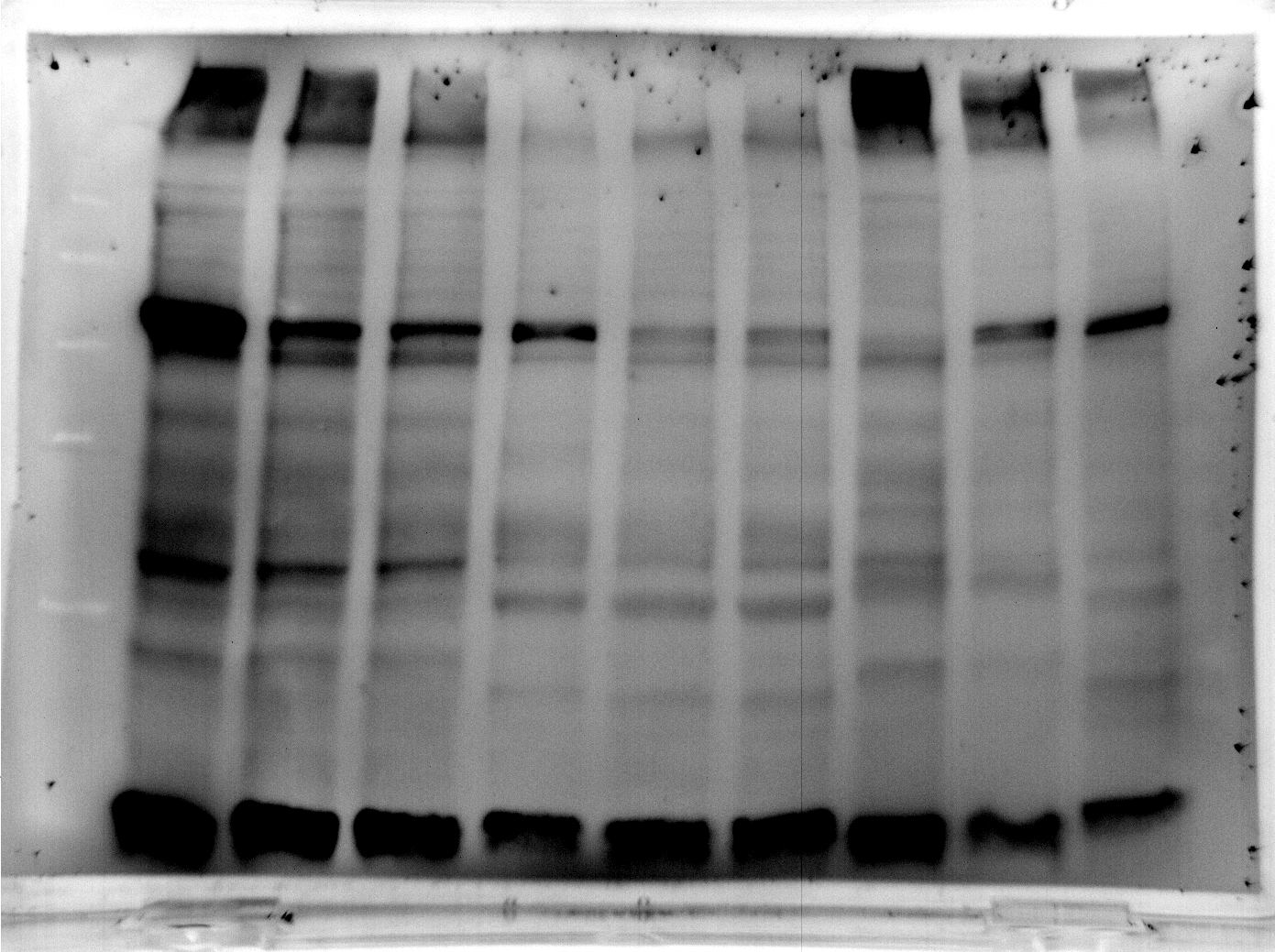

Supplement: Figure 1—figure supplement 1—source data 2. — The membrane was first developed using p130cas antibody (A) and then using GAPDH antibody (B). [file elife-96157-fig1-figsupp1-data2.zip › Figure 1- figure supplement 1- source data 2/Figure 1- figure supplement 1- source data 2 B.tif]

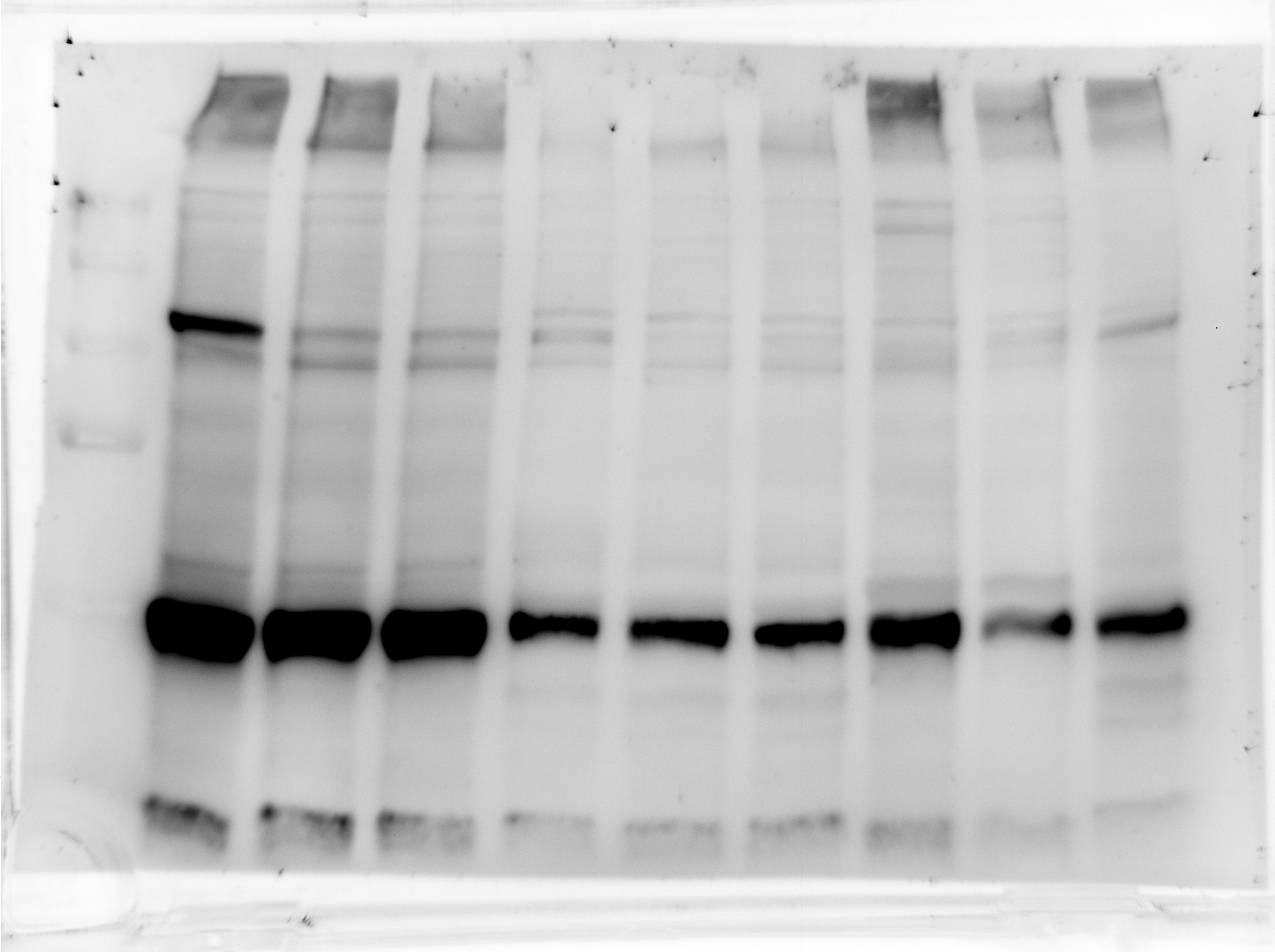

Supplement: Figure 7—source data 1. — The membrane was first developed using p130cas antibody (A) (and then using the GAPDH antibody) and then using the tubulin antibody (B). [file elife-96157-fig7-data1.zip › Figure 7- source data 1/Figure 7- source data 1 B.tif]

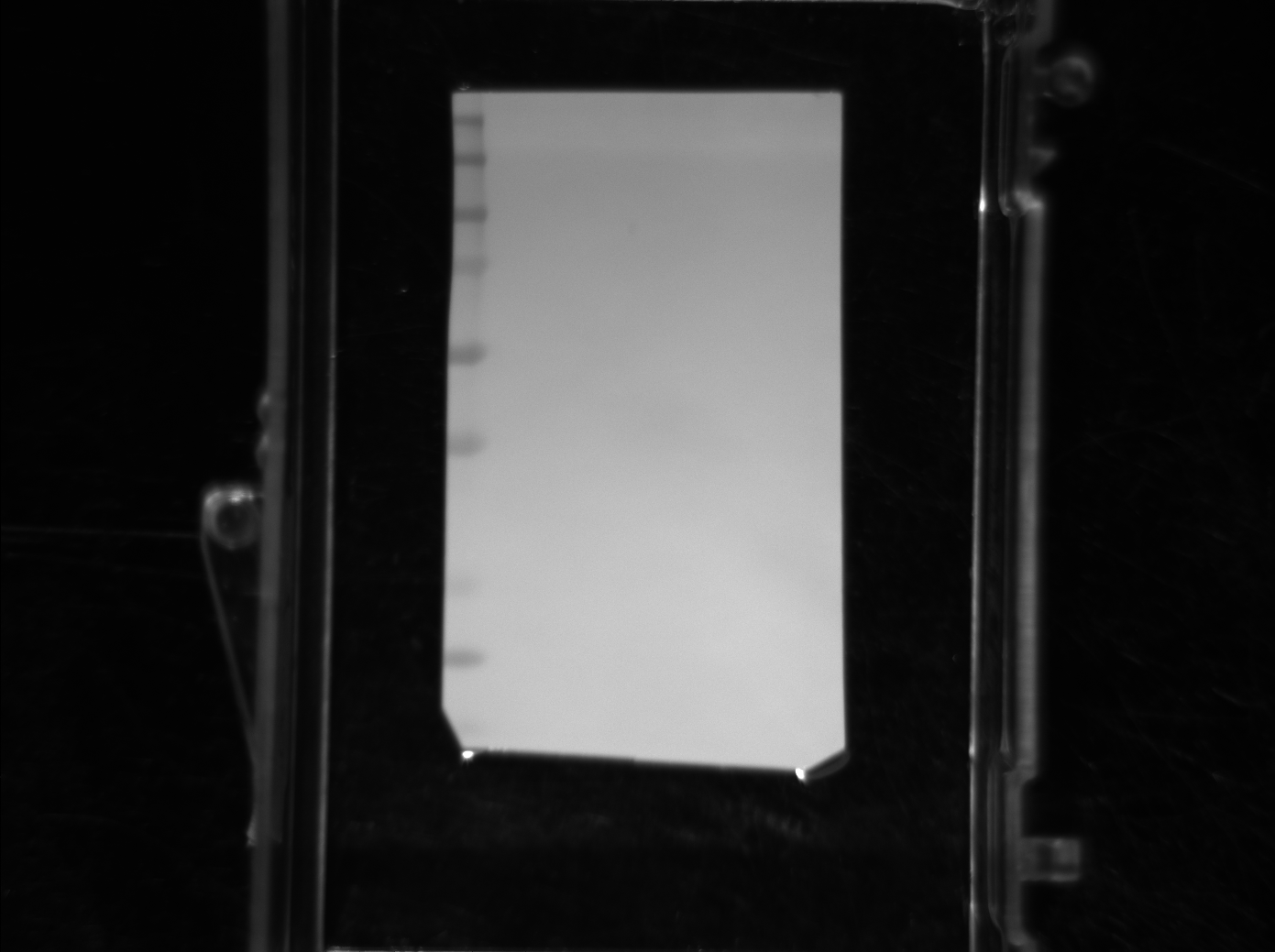

Supplement: Figure 7—source data 2. — The membrane was first developed using p130cas antibody (A) and then using the GAPDH antibody (B). White light image corresponding to each membrane is shown in the right panel. [file elife-96157-fig7-data2.zip › Figure 7- source data 2/Figure 7- source data 2 A- ladder.tif]

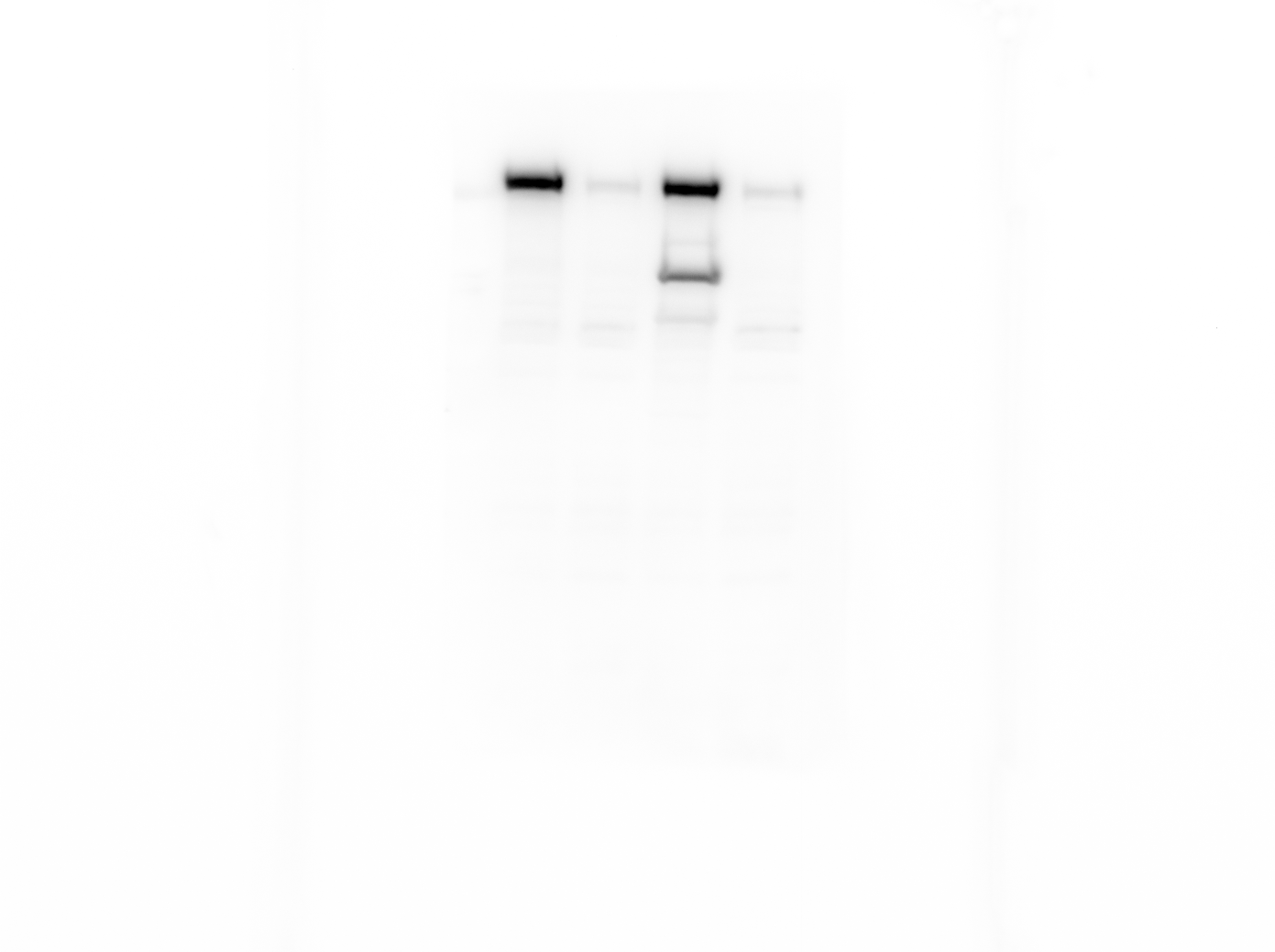

Supplement: Figure 7—source data 2. — The membrane was first developed using p130cas antibody (A) and then using the GAPDH antibody (B). White light image corresponding to each membrane is shown in the right panel. [file elife-96157-fig7-data2.zip › Figure 7- source data 2/Figure 7- source data 2 A.tif]

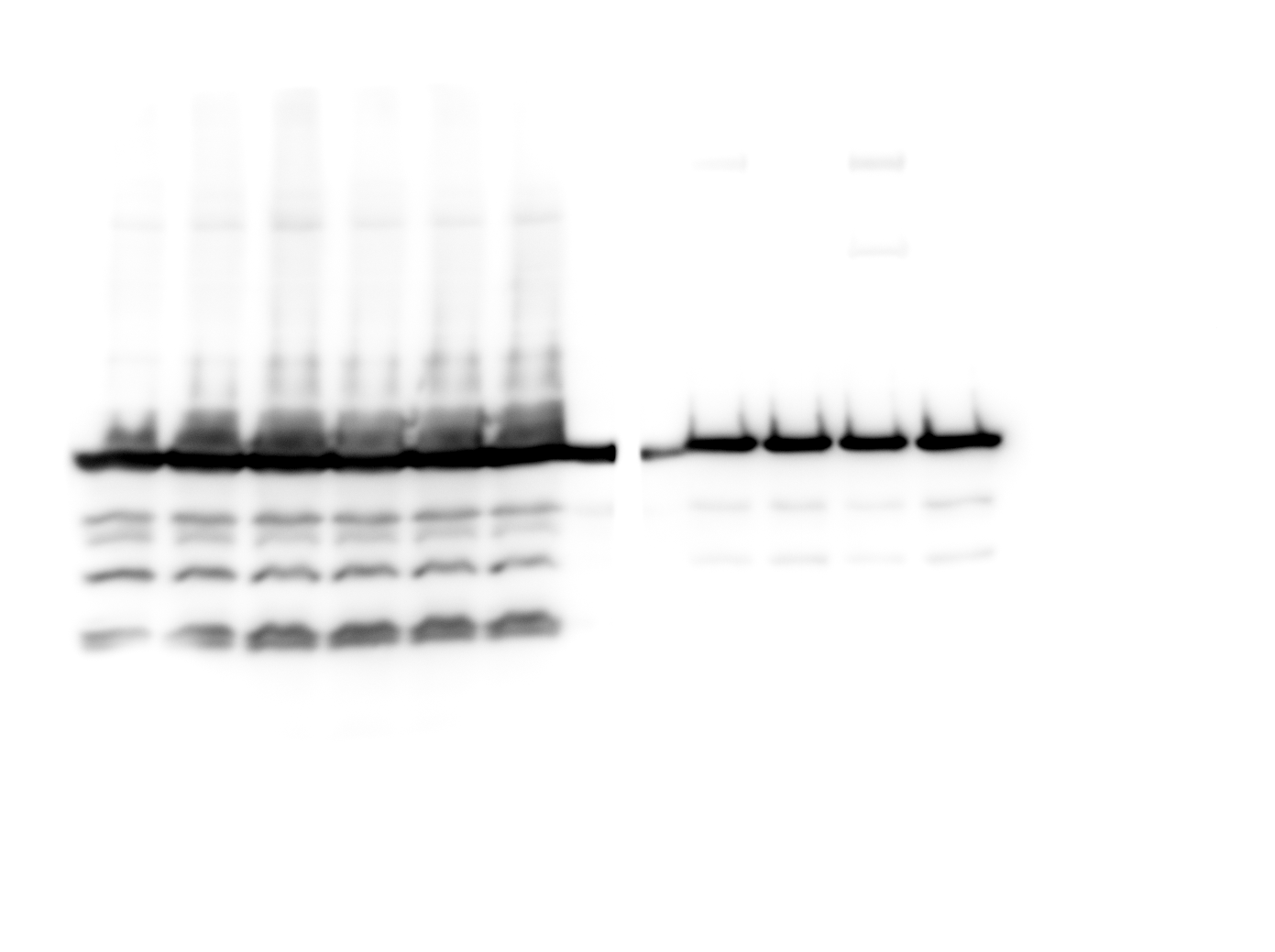

Supplement: Figure 7—source data 2. — The membrane was first developed using p130cas antibody (A) and then using the GAPDH antibody (B). White light image corresponding to each membrane is shown in the right panel. [file elife-96157-fig7-data2.zip › Figure 7- source data 2/Figure 7- source data 2 B.tif]

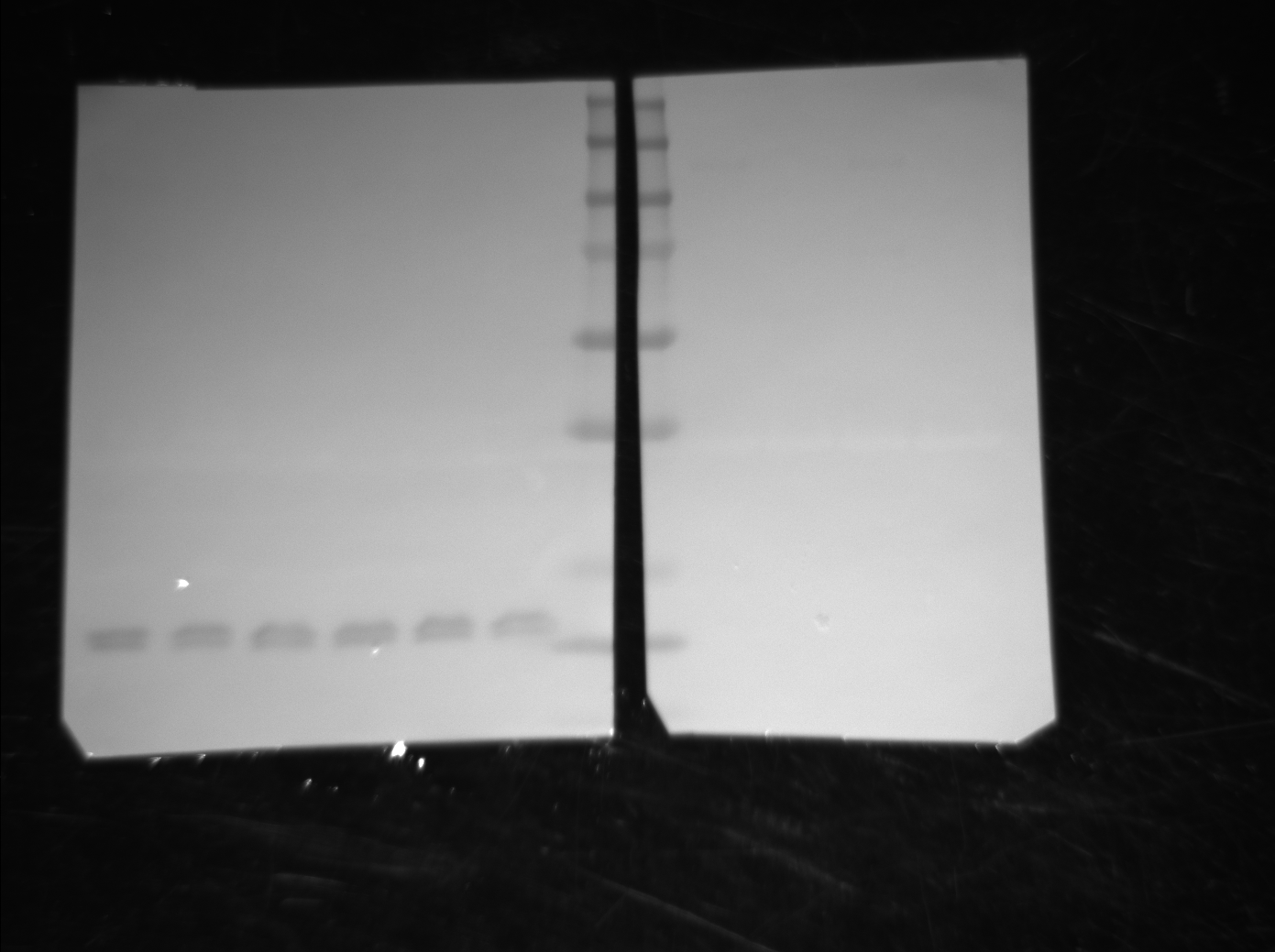

Supplement: Figure 7—source data 2. — The membrane was first developed using p130cas antibody (A) and then using the GAPDH antibody (B). White light image corresponding to each membrane is shown in the right panel. [file elife-96157-fig7-data2.zip › Figure 7- source data 2/Figure 7- source data 2 B-ladder.tif]

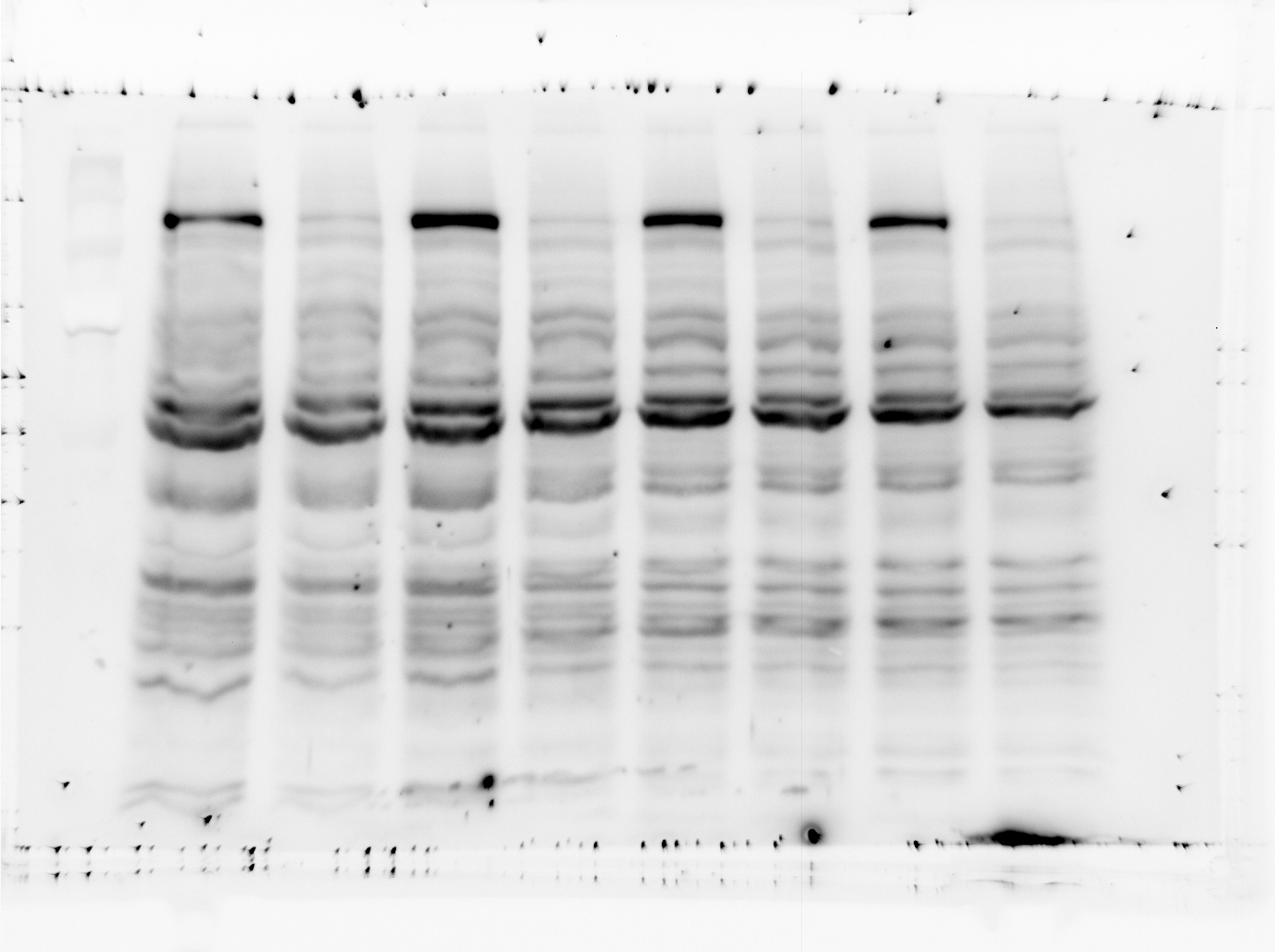

Supplement: Figure 7—figure supplement 1—source data 1. — The membrane was first developed using p130cas antibody (A) and then using GAPDH antibody (B). [file elife-96157-fig7-figsupp1-data1.zip › Figure 7- figure supplement 1- source data 1/Figure 7- figure supplement 1- source data 1 A.tif]

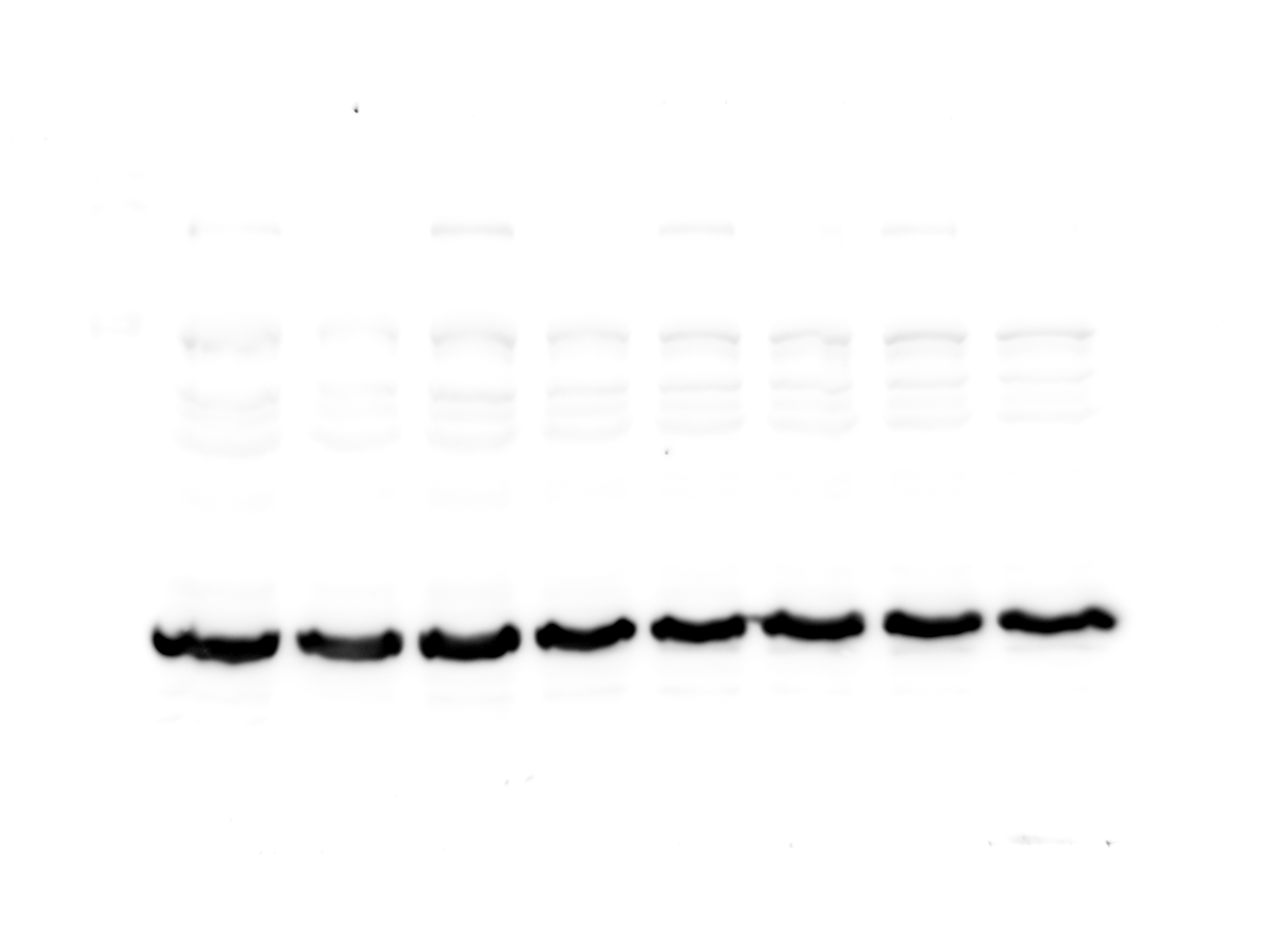

Supplement: Figure 7—figure supplement 1—source data 1. — The membrane was first developed using p130cas antibody (A) and then using GAPDH antibody (B). [file elife-96157-fig7-figsupp1-data1.zip › Figure 7- figure supplement 1- source data 1/Figure 7- figure supplement 1- source data 1 B.tif]

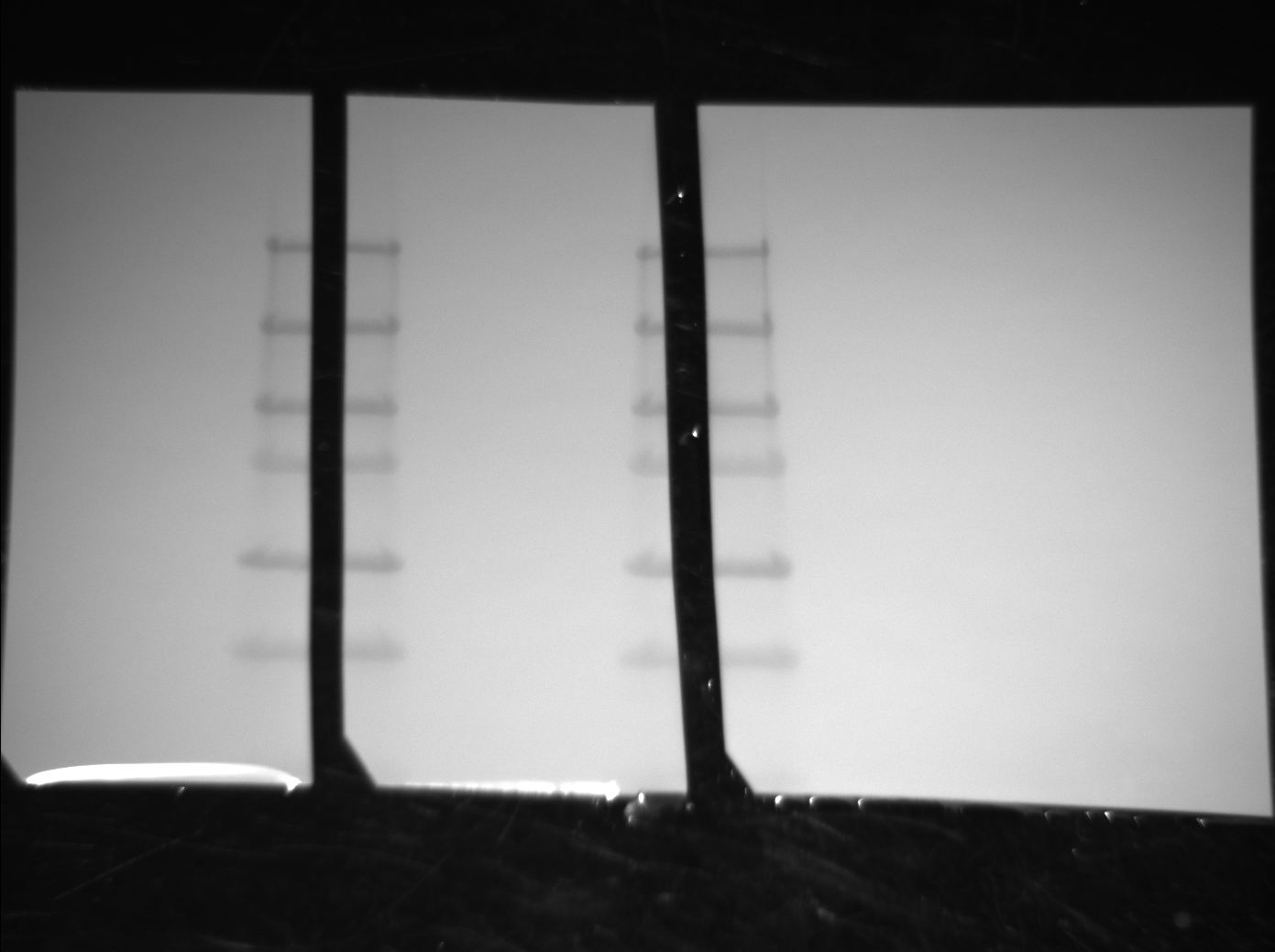

Supplement: Figure 8—figure supplement 1—source data 1. — The membrane was first developed using p130cas antibody (A) and then using GAPDH antibody (B). White light image corresponding to each membrane is shown in the right panel. [file elife-96157-fig8-figsupp1-data1.zip › Figure 8- figure supplement 1- source data 1/Figure 8- figure supplement 1- source data 1 A ladder.tif]

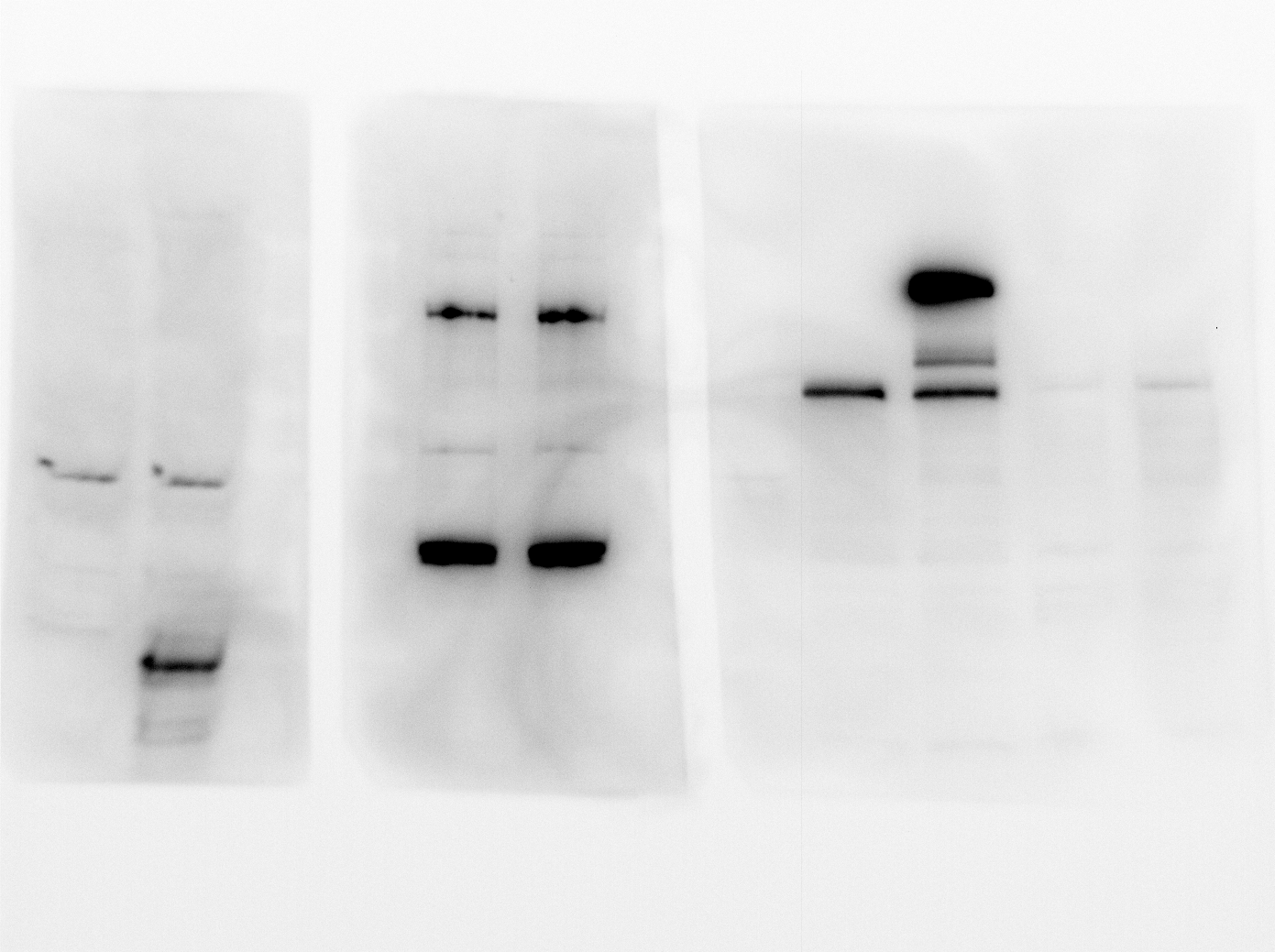

Supplement: Figure 8—figure supplement 1—source data 1. — The membrane was first developed using p130cas antibody (A) and then using GAPDH antibody (B). White light image corresponding to each membrane is shown in the right panel. [file elife-96157-fig8-figsupp1-data1.zip › Figure 8- figure supplement 1- source data 1/Figure 8- figure supplement 1- source data 1 A.tif]

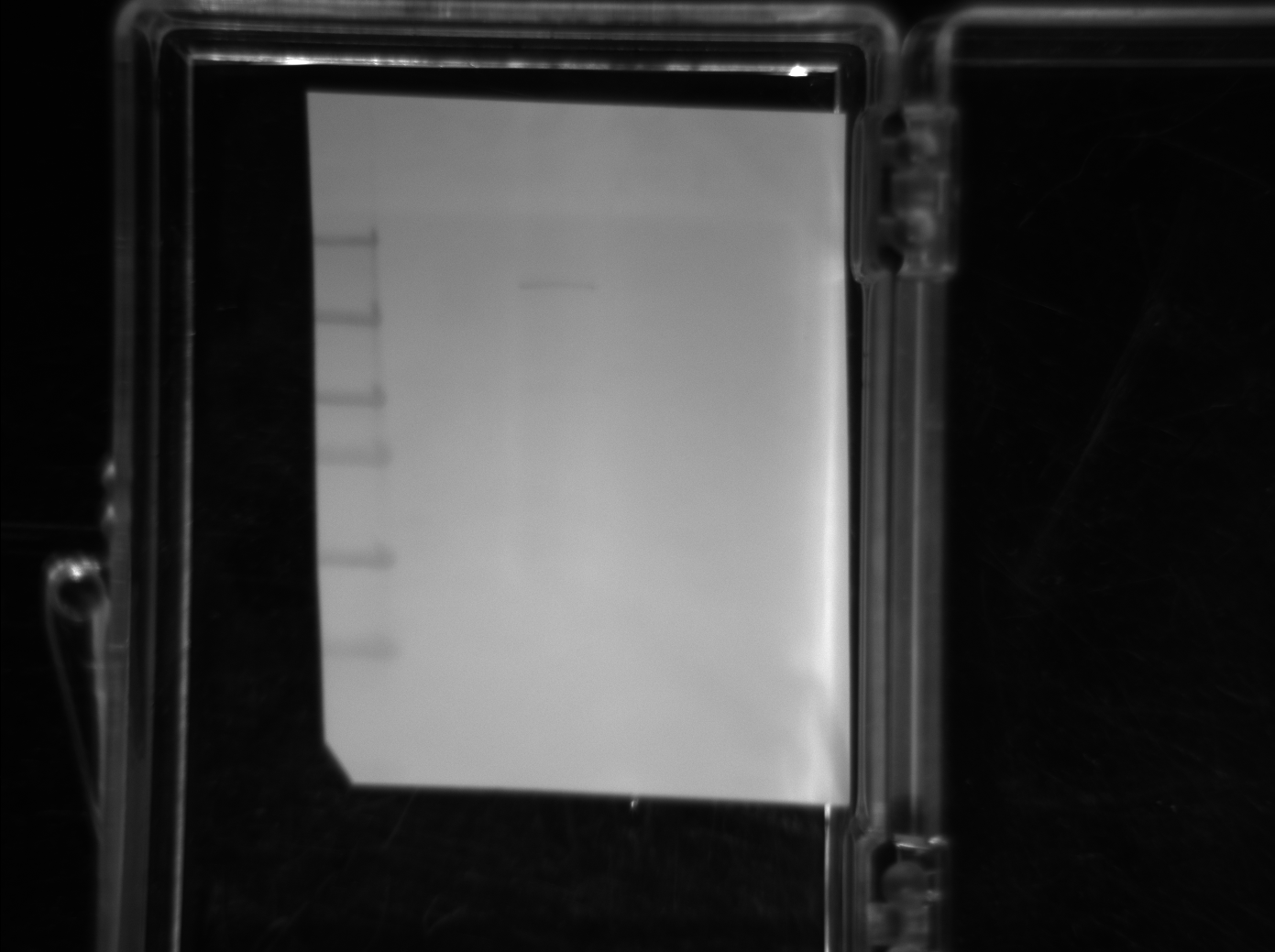

Supplement: Figure 8—figure supplement 1—source data 1. — The membrane was first developed using p130cas antibody (A) and then using GAPDH antibody (B). White light image corresponding to each membrane is shown in the right panel. [file elife-96157-fig8-figsupp1-data1.zip › Figure 8- figure supplement 1- source data 1/Figure 8- figure supplement 1- source data 1 B ladder.tif]

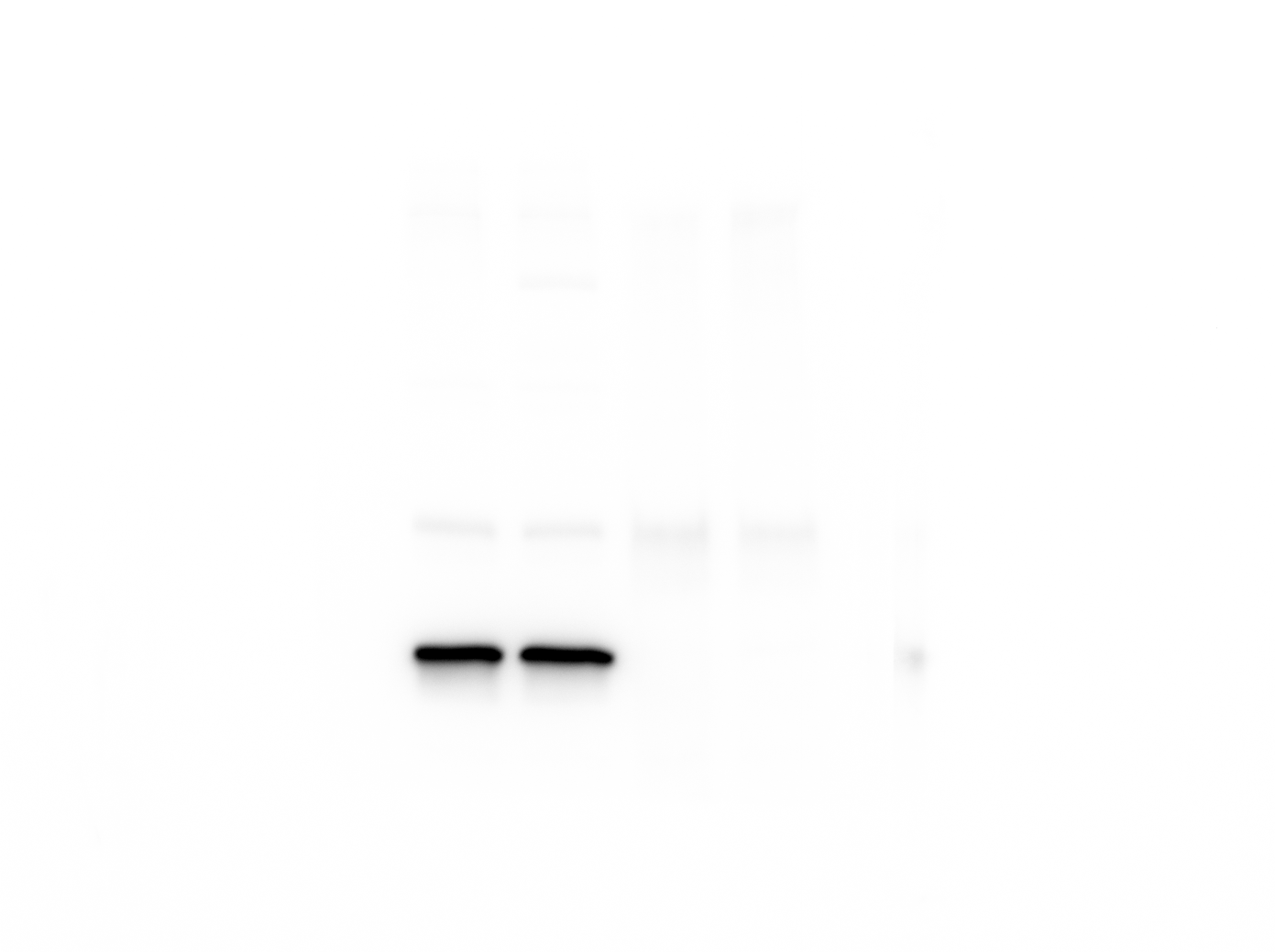

Supplement: Figure 8—figure supplement 1—source data 1. — The membrane was first developed using p130cas antibody (A) and then using GAPDH antibody (B). White light image corresponding to each membrane is shown in the right panel. [file elife-96157-fig8-figsupp1-data1.zip › Figure 8- figure supplement 1- source data 1/Figure 8- figure supplement 1- source data 1 B.tif]
